# The Effect of a Maternal Double Megadose of Vitamin A Supplement on Serum Levels of Retinol in Children Aged under Six Months

**DOI:** 10.1155/2013/876308

**Published:** 2013-12-24

**Authors:** Carmina Silva dos Santos, Ilma Kruze, Taciana Fernandes, Luciana Marques Andreto, José Natal Figueiroa, Alcides da Silva Diniz

**Affiliations:** ^1^Departamento de Nutrição, Centro de Ciências da Saúde, Universidade Federal de Pernambuco, 50670-901 Recife, PE, Brazil; ^2^Departamento de Pesquisa, Instituto de Medicina Integral Prof. Fernando Figueira (IMIP), 50070-550 Recife, PE, Brazil

## Abstract

*Objective*. To measure concentrations of serum retinol in children after the use of maternal vitamin A double megadose supplements. *Design*. Randomized controlled clinical trial. *Setting*. The study was conducted at two maternity hospitals in the city of Recife, in the northeast region of Brazil between August 2007 and June 2009. *Subjects and Methods*. 276 children/mothers were recruited after birth and the women received a 200,000 IU capsule of vitamin A. After ten days they were randomly assigned to two treatment groups. One group received a second 200.000 IU capsule, while the other received a placebo. The concentrations of retinol in the serum of the children from each group were measured at 2, 4, and 6 months. *Results*. 173 children completed the study. There was no difference between the two treatment groups (*P* = 0.514). The mean base retinol level was lower than that at four and six months (*P* < 0.001). *Conclusions*. The maternal double megadose supplement had no additional effect on the serum retinol levels of the children, although concentrations of retinol in the children rose in the first six months of life. This trial is registered with NCT00742937.

## 1. Introduction

Vitamin A deficiency (VAD) is considered to be a serious problem in more than seventy countries, and, according to the World Health Organization (WHO), around 2.8 million children of preschool age are clinically affected by the condition [1]. Deficiency may therefore lead to repercussions, such poor night vision, if the deficiency is mild, which may develop into irreversible blindness, as well as stunting the growth of the child and diminishing resistance to infection [2, 3].

It is known that breastfeeding infants aged under six months are considered a group at risk of vitamin A deficiency. This is due to the fact the reserves of vitamin A in the liver of the breastfeeding child are very limited at birth. This occurs for the following reasons: a tendency for levels of serum retinol to be lower in pregnant women and the existence of a selective placental barrier, which blocks the passage of this vitamin to the fetus [4].

Hence, the use of a megadose of vitamin A for children aged between 6 and 59 months in areas at risk of vitamin A deficiency is one of the main short-term interventions for combating this deficiency in the field of public health. The use of vitamin A supplements in this areas helps to reduce mortality in children aged under five years by 30% and can diminish the severity of diarrheal diseases [5, 6]. Breastfeeding thus provides great protection for children aged up to two years, which is the age during which they are most at risk of damage [7].

The vitamin A content in human milk is variable and influenced by several factors such as age, parity, and socioeconomic status of the mother, postpartum age, volume, and fat content of the milk. The concentration of vitamin A in the colostrum is high (>331 IU/dL or >99 mcg/dL) and decreases gradually in the first months of lactation, whereas mature milk concentration ranges from 110 to 257 IU/dL (33 to 77 mcg/dL) [7].

Strategies for improving the vitamin A status of breastfeeding infants and children of school age have been proposed, given that breastfeeding is the best way to protect children up to two years of age. In 2002, the International Vitamin A Consultative Group (IVACG) recommended the use of higher doses of vitamin A (400,000 IU) during the immediate postpartum period as a safer way of preventing vitamin A deficiency during breastfeeding, although current evidence is insufficient to back up this proposal [8].

Thus, this study aims to measure the concentrations of serum retinol after two, four, and six months in children of mothers who received a double megadose of vitamin A during postpartum and to assess the protective effect of this vitamin A supplement in terms of maintaining adequate serum levels of retinol in breastfeeding children.

## 2. Methods

### 2.1. Design and Study Setting

A controlled triple-blind randomized clinical hospital-based trial was carried out with women and their progeny attending the Instituto de Medicina Integral Prof. Fernando Figueira (IMIP) and Bandeira Filho maternity hospitals between August 2007 and June 2009.

### 2.2. Sample Size

The sample size was calculated using the formula: *N* = (*u*+*v*)^2^ (*dp*_1_^2^ + *dp*_2_^2^)/(*μ*_1_−*μ*_2_)^2^ [9] supposing error *α* = 5% (*u*), error *β* = 20% (*v*), a standard deviation of the distribution of concentrations of serum retinol, in the two groups (*dp*_1_ and *dp*_2_) of 0.5 *μ*mol/L, and a difference between the mean retinol levels between the groups (*μ*_1_−*μ*_0_)^2^ of 0.25 *μ*mol/L, giving a minimum sample size for each group of 73 children. So as to cover for any losses, a sample of 146 was taken for each group.

### 2.3. Study Population

The study sample comprised 293 newborns from women with a low obstetric risk pregnancy admitted to the IMIP and Bandeira Filho maternity, in the municipality of Recife, who agreed to participate by signing a Term of Free and Informed Consent. Newborns were excluded if their mothers had psychiatric disorders, if they had severe perinatal hypoxia (apgar < 4 in the 1st and 5th minutes of life), or if they had malformations and/or other serious illnesses that are known to be able to affect susceptibility to infectious diseases or growth, to make it difficult to measure weight and height, or to prevent breastfeeding (malabsorption syndromes, metabolic syndromes, cardiopathy, and cleft palette).

*Selection/Randomization and Followup of Participants*. The women were selected when they were admitted to hospital for the birth, on which occasion they were informed of the aims of the research and responded to a structured questionnaire, to collect sociodemographic, clinical, biochemical, anthropometric, and nutritional data.

Shortly after birth, blood was collected from the umbilical cord, weight, height, and the circumference of the head were measured, and each mother received a 200,000 IU vitamin A capsule (Farmanguinhos/FIOCRUZ, Rio de Janeiro, RJ, Brasil), according to the protocol recommended by the Brazilian Ministry of Health.

Unable to deploy one control group without administration of the first capsule of vitamin A, because according to the supplementation program of the Ministry of Health of Brazil capsule of vitamin A is provided to all postpartum women in public hospitals.

The 10th day after-partum, during the consultation, the individual randomization was carried out by the pediatric nurse, using a table of random numbers generated by the EPI INFO program Version 6.04 d (WHO/CDC, Atlanta, GE, USA). The children and their mothers were randomly assigned to two groups (group 1 and group 2): in group 1, the mothers received a second vitamin A capsule (200,000 IU) making a total of 400,000 IU, and, in group 2, the mothers received a placebo capsule, making a total of 200,000 IU of vitamin A. The randomization codes were kept secret during the whole study and were revealed only after the conclusion of data analysis.

On the three days following receipt of the supplement, all the women and children were monitored for side effects arising from the toxicity of vitamin A, when administered in high doses. Nothing was mentioned to the mothers that could be related to these side effects in order to avoid suggestibility to some of the symptoms under study.

**Figure 1:**
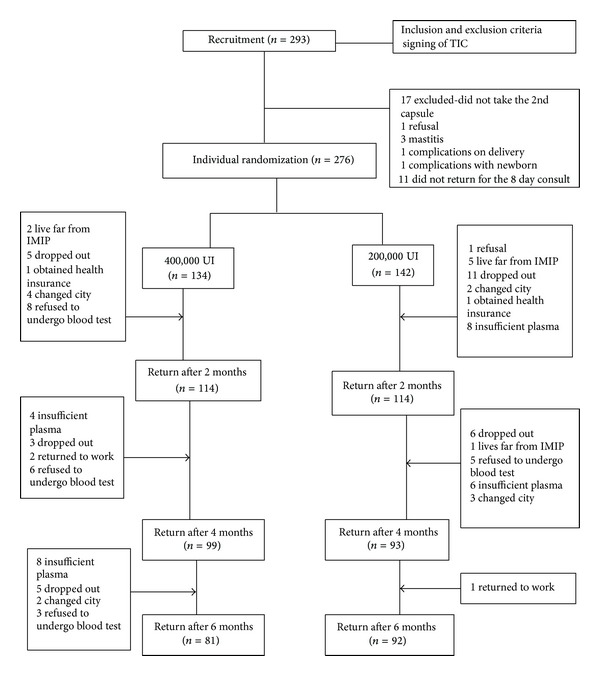
Flowchart of followup.

The flowchart for selection, randomization, and followup/losses of participants is shown in Figure 1.

### 2.4. Details of the Groups

 Group 1: the mother received a 200,000 IU capsule (retinyl palmitate) +40 mg of vitamin E, administered orally, and shortly after birth and 10 days after-partum, a second 200,000 IU capsule (retinyl palmitate) +40 mg of vitamin E was administered.
 Group 2: the mother received a 200,000 IU capsule (retinyl palmitate) +40 mg of vitamin E, administered orally, and shortly after birth and 10 days after-partum, a second “placebo” capsule containing 40 mg of vitamin E dissolved in soya oil.

The vitamin A and placebo capsules were prepared in such a way as to be identical in size, shape, color, and taste by Relthy Laboratórios (Indubatuba, SP, Brasil). All the capsules contained vitamin E, both to avoid possible toxic side effects of high doses of vitamin A and to give the capsule more stability.

### 2.5. Methods and Measurement Techniques

In children, all blood samples were taken at the same time of day, between 8 and 10 am, by a duly trained technician. At the consultations in the 2nd and 4th month after-partum, 3 mL of blood was collected by venocubital punction, and at the consultation in the 6th month, a further 2 mL was collected for the hemogram.

The aliquots used for analysis of concentrations of serum retinol were placed in tubes protected from the light and the 2 mL aliquots for analysis of hemoglobin were placed in a tube containing the anticoagulant ethylenediamine tetraacetic acid (EDTA).

In the case of the analysis of concentrations of serum retinol, one hour after decanting, the samples were spun in a centrifuge at 3000 rpm for 10 minutes, and the serum was separated and placed in Eppendorf tubes, stored in a freezer at −20°C, and then transported to the Centro de Investigação em Micronutrientes (CIMICRON) of the Universidade Federal da Paraíba (UFPB), in a cooler. The concentrations of serum retinol were analyzed using high resolution liquid chromatography (HPLC, model 305, Gilson, France), according to the technique established by Tanumihardjo et al. [10]. The cut-off point adopted for identification of serum retinol deficiency was <0,70 *μ*mol/L [11].

The socioeconomic variables and the nutritional habits of the mother and child were obtained during an interview at the hospital. Information on per capita family income was expressed in terms of minimum wages (MW) and divided into three groups: <1/4 MW (<US$ 62.2), 1/4 | −1/2 MW (US$ 62.2–124.4), and ≥1/2 MW (≥US$ 124.4) (1 MW = US$ 248.8).

The level of schooling of the mother was expressed in terms of years of schooling and divided into the following categories: <8 years, 8 | −11 years and >11 years. Sanitation was considered to be adequate when there was a connection to the local sewerage network, regular trash collection, and water available from the public network through indoor piping and inadequate when one of these was absent.

The mother's vitamin A intake was assessed using the previously validated Quantitative Nutrition Frequency questionnaire, adapted for the foodstuffs common in the region. This questionnaire was applied with the help of colored photos of utensils and food [12, 13] in order to obtain greater precision as to the quantities ingested. This questionnaire was administered at the end of the trial and its takes into consideration the food consumption referring to the intake of different types of food in previously defined small, medium, or large portions, eaten daily, weekly, or monthly. The questionnaire measures food intake according to portions (small, medium, or large) per week, per day, or per month. The diets were analyzed using the DietSys program Version 4.01 (National Cancer Institute, Bethesda, MD, USA) which is based on the Table of the Chemical Composition of Foodstuffs drawn up by the US Department of Agriculture (USDA), and there were no mothers with inadequate intake of vitamin A.

Child nutrition was classified according to the categories recommended by the WHO as follows: exclusive maternal breastfeeding: children who exclusively drink their mother's milk, except for the use of medication and mineral and vitamin supplements; maternal breastfeeding: children who drink mother's milk, cow's milk, and other food. The measurement of weight and height was carried out in duplicate by research assistants with prior training by the research team and the measurements for every fifth child were taken again by one of the researchers as a way of ensuring intra- and interobserver reliability, as recommended by Frisancho [14]. Weight was measured using Fillizola, E-150/3P model microelectronic scales and height using a wooden ruler. An error of 100 g and 0.1 cm, respectively, was accepted for weight and height [15]. Anthro software Version 2 was used to calculate and analyze the anthropometric data.

### 2.6. Statistical Analysis

The data were entered in duplicate and confirmed using the “Validate” module of the Epi-info statistics package, Version 6.04 d (WHO/CDC, Atlanta, GE, USA), to check consistency and provide validation. Statistical analysis was carried out using the Statistical Package for Social Sciences (SPSS) for Windows, Version 12.0 (SPSS Inc., Chicago, IL, USA) and STATA (Statacorp, College Station, TX, USA).

The variables that presented a normal distribution after application of the KOLMOGOROV-SMIRNOV test were described in terms of mean and standard deviation. The only variable to present a nonnormal distribution was nutrition and this was described in terms of median and interquartile range. For comparison of the means for the two groups, Student's *t*-test was used for nonpaired data and for comparison of the proportions, Pearson's chi-squared and Fisher's exact tests were employed. Analysis of variation in retinol by time and dosage was adjusted for linear regression models using the generalized estimation equations (GEE) technique, which adjusts the estimates of the parameters and their standard errors, taking into consideration the possible correlation of the repeated measures. To avoid bias in estimating the temporal variation of retinol after treatment, a variable indicating the base measurements was introduced into the regression models.

## 3. Results

The random distribution resulted in a homogeneous sample both in terms of the characteristics of the mothers and the children (Table 1). Most were found to have an income of more than half one minimum wage, their homes had adequate sanitation and most were young adults with more than eight years of schooling.

Of the 293 children recruited for the study, 173 completed the protocol. The dropout rate was around 38%, although these losses did not affect the characteristics of the groups of remaining individuals (*P* = 0.08) (Tables 2 and 3).

The feeding pattern was similar in the two supplement groups throughout the follow-up period. (Table 4).

The adjustment to the linear regression model using the GEE technique suggested that the serum concentrations of retinol were similar in the two supplement groups throughout the six-month follow-up period (*P* = 0.514). There was no significant interaction between the treatment group and the time of followup (*P* = 0.693) (Table 5).

There was no statistically significant difference between the two groups in terms of mean serum retinol at the baseline and after two months (*P* = 0.484) and the same was true for the fourth and sixth months (*P* = 0.421). The mean retinol levels at the baseline were significantly lower than the mean after four months (*P* < 0.001) and after six months (*P* < 0.001). The mean levels of retinol after two months were significantly lower than the mean after four months (*P* < 0.001) (Table 6).

**Figure 2:**
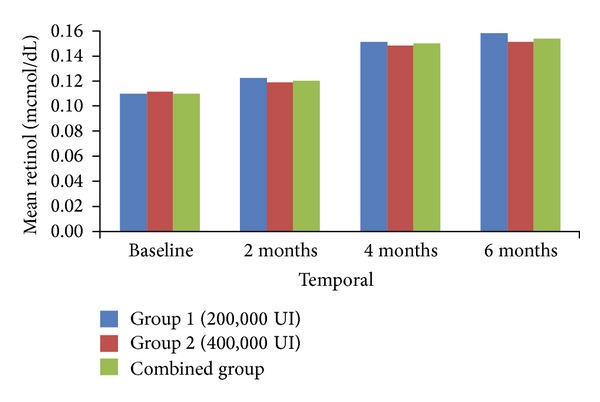
Mean retinol, by treatment group and duration of followup.

Figure 2 shows that serum concentrations of retinol were similar in the treatment group and the combined group.

## 4. Discussion

Few studies have evaluated the use of vitamin A supplements by mothers, either in a megadose [16–18] or in higher doses [9, 19–23], and the serum concentrations in children at various points during a follow-up period.

Our data show that over time there was no significant interaction between the treatment groups and no difference was found between the groups receiving a supplement of 400,000 IE and those receiving a dose of 200,000 IU. The expected increase after taking a higher dose of supplement may fail to appear in serum levels, since these do not necessarily reflect the liver reserves of this vitamin, and it is known that there is a serum retinol limit as an absolute measure to establish the vitamin A status of an individual, given that these levels are controlled homeostatically and their concentrations should be altered at times when liver reserves are low or in situations compatible with vitamin A deficiency A.

Our findings are backed up by studies carried out in Tanzania [22] which used high doses of vitamin A supplements and found no difference between the treatment groups so far as serum concentrations of this micronutrient are concerned. However, Ayah et al. [23] have noted that supplements in a double megadose do not appear to have an impact on serum levels, but to increase liver reserves in the children, as was not proved by Idindili et al. in Tanzania [22].

A study conducted in Ghana showed that the 400,000 IU dose of supplement in women probably increased the liver reserves more than the 200,000 IU dose, although the modified relative dose respond (MDRD) test was not capable of showing this. Base on this observation, Tchum et al. proposed the use of the stable isotope method in future research, as a way of improving the quantitative evaluation of concentrations of retinol in the liver, which might show the possible benefits of vitamin A supplements in higher doses [20].

So far as liver reserves are concerned, it was not possible to measure this in the population studied and this may be considered a limitation of this study, which restricted itself to concentrations of vitamin A in serum. However, the concentration of retinol in serum may not be the most appropriate indicator for evaluating the effect of these dosages, although it is the indicator recommended by the WHO and the IVACG for investigation of the organic status of vitamin A.

The data also point to a growing increase in base levels up to the sixth month. One plausible explanation of this would be that the liver reserves of vitamin A at birth are highly limited and this is because of the existence of a selective barrier in the placenta, which controls the passage of this vitamin to the fetus, as a way of preventing possible teratogenic effects [4].

It may be that once effective breastfeeding has been established, it is possible to build up reserves of this micronutrient in the child and it should be pointed out that most breastfed for the first six months of their life, albeit not exclusively, as it should be to ensure that this population has a satisfactory intake of this micronutrient, because there was no significant difference between children who received exclusive breastfeeding and those artificially fed (unpublished data).

This is even more indispensable in the first few days of breastfeeding, since colostrum plays a fundamental role in the initial formation of stocks of vitamin A in the liver of the breastfeeding infant, before serum levels of retinol begin to decline [24].

Another fact that could explain these growing concentrations is the nutritional status of the children studied, who mostly had an age-appropriate BMI and did not have frequent episodes of infection (data not published), with a consequent reduction in deprivation, which may be reflected in their vitamin A status.

Our results may be compared to a study [18] in an Indian population, which showed that when mothers received a single megadose of supplement, there was a statistically significant difference between the concentrations of retinol in the children at the baseline and after five months.

However, this has not been found by other studies which also used a double megadose of vitamin A, although these do not show a retinol curve for children during a follow-up period after receiving the supplement [21–23].

However, we should stress that although our population lives in an areas where vitamin A deficiency is endemic, this population has a distinct profile of poverty, although there is not extreme poverty. Likewise the study was not based on the population as a whole and it is therefore difficult to make inferences about other populations.

The importance of the data presented here stems from the fact that there are no studies in the literature that report concentrations of serum retinol at more than two stages in the life of children aged under six months and back the need to maintain the WHO recommended maternal supplements as a fairly effective strategy for combating vitamin A deficiency, in combination with the promotion of breastfeeding, when 60 times more vitamin A is transferred to the child, in view of the fact that the supplement can be administered in a single visit by the mother to the health services, while, for a child, it would require numerous visits.

However, the data presented here should be interpreted with caution. A first attempt would be concern regarding the national “More Vitamin A” program's distribution at six and twelve months, which should perhaps occur at a later stage.

It can be concluded that additional effects were not detected on the serum concentrations of retinol in children after mothers had taken a double megadose of vitamin A supplement during postpartum, contrary to the current recommendations of the WHO, although concentrations of retinol in the children tended to grow in the first six months of life.

## Figures and Tables

**Table 1:** Baseline characteristics of mothers and children, Recife, Brazil, 2008-2009.

Characteristics	400,000 IU vitamin A			200,000 IU vitamin A			P*
	*n*	Mean	SC	*n*	Mean	SD	
Children							
Male [*n*(%)]	134	65	(48.5)	142	5568	(47.9)	0.69^†^
Weight at birth (g)	134	3344.0	405.3	142	3283.0	373.1	0.24
Length (cm)	134	48.9	2.0	142	48.9	1.8	0.86
Circumference of cranium (cm)	133	34.5	1.7	141	34.6	2.1	0.70
Serum retinol (*µ*mol/L)	26	1.09	0.5	27	1.11	0.5	0.93
Breastfeeding^§^[*n* (%)]	115	94	(81.7)	109	77	(70.6)	0.07
Mothers							
Age (years)	134	24.3	5.6	142	24.9	6.8	0.48
Schooling (years)	134	8.8	2.9	142	8.8	2.5	0.86
Per capita income ($^∣∣^)	130	89.4	63.3	132	83.6	47.7	0.38
Adequate sanitation [*n* (%)]^¶^	127	28	(56.0)	123	22	(44.0)	0.42^†^
Vitamin A intake** (*µ*g) [Med; IQ^††^]	69	1090.4	817.7–1648.5	72	1164.7	841.0–1748.7	0.76^‡^
Serum retinol (*µ*mol/L)	106	1.6	0.7	106	1.7	0.8	0.55

*Student's *t*-test for nonpaired data; ^†^chi-squared test; ^‡^Mann Whitney *U*-test; ^§^maternal breastfeeding in the 6th month of life; ^∣∣^$: US dollars; ^¶^inadequate sanitation: household without one or more of the following characteristics: indoor piped water, regular trash collection, and link to the sewerage network; **vitamin A intake in *µ*g of retinol; ^††^IQ: interquartile range.

**Table 2:** Distribution of children dropping out by supplement group, Recife, PE, 2007/09.

Supplement group	Study		Dropout rate		Total		P*
	*n*	%	*n*	%	*N*	%	
400,000 IU vitamin A	115	85.8	19	14.2	134	100.0	0.08
200,000 IU vitamin A	109	76.8	33	23.2	142	100.0	

*χ^2^-test *P* = 0.08.

**Table 3:** Comparison of characteristics of mothers and children followed up by the study with those who dropped out, Recife, PE, 2007/09.

Characteristics	Study		Drop outs		P*
	*n*	Mean ± SD	*n*	Mean ± SC	
Mothers					
Age (years)	224	24.6 ± 6.2	52	23.6 ± 5.5	0.22
Schooling (years)	224	8.8 ± 2.7	52	8.4 ± 2.4	0.27
Per capita income (R$)	222	145.3 ± 97.2	49	133.8 ± 69.0	0.37
BMI (kg/m^2^)	176	28.5 ± 4.4	29	27.2 ± 4.6	0.08
Children					
Birth weight (g)	224	3313.0 ± 390.0	52	3283.0 ± 415.4	0.57
Males [*n* (%)]	224	107 (47.8)	52	26 (50.0)	0.38
Adequate sanitation [*n* (%)]	221	171 (77.5)	30	25 (83.3)	0.32

*Student's *t*-test for unpaired data.

**Table 4:** Distribution of maternal breastfeeding in children after six months by supplement group, Recife, 2008-2009.

Type of feeding	400,000 IU vitamin A		200,000 IU vitamin A		P*
	*n*	%	*n*	%	
2 months					
Exclusive maternal breastfeeding	70	52.24	64	47.76	0.48
Maternal breastfeeding	38	46.34	44	53.66	
4 months					
Exclusive maternal breastfeeding	50	54.35	42	45.65	0.76
Maternal breastfeeding	52	57.14	39	42.86	
6 months					
Exclusive maternal breastfeeding	20	21.7	23	29.1	0.29
Maternal breastfeeding	72	78.3	56	70.9	
Total	**92**	**100**	**79**	**100**	

*Chi-squared test.

**Table 5:** Distribution of means for retinol by duration of followup and supplement group, Recife, 2008/2009.

Vitamin A group	Time			
	Baseline mean ± SD (*n*) (*µ*mol/L)	Two months mean ± DP (*n*) (*µ*mol/L)	Four months mean ± DP (*n*) (*µ*mol/L)	Six months mean ± DP (*n*) (*µ*mol/L)
200,000 IU	1.09 ± 0.50 (25)	1.22 ± 0.66 (114)	1.51 ± 0.67 (99)	1.57 ± 0.60 (81)
400,000 IU	1.11 ± 0.55 (28)	1.18 ± 0.57 (114)	1.48 ± 0.55 (93)	1.51 ± 0.53 (92)

Combined group	1.10 ± 0.52 (53)	1.20 ± 0.62 (228)	1.50 ± 0.61(192)	1.54 ± 0.56 (173)

**Table 6:** Estimated*mean serum retinol in combined group, during followup, Recife, 2008/2009.

	Time			
	Baseline mean (CI_95%_) (*µ*mol/L)	2 months mean (CI_95%_) (*µ*mol/L)	4 months mean (CI_95%_) (*µ*mol/L)	6 months mean (CI_95%_) (*µ*mol/L)
Combined group	1.14 (1.00–1.29)	1.20 (1.12–1.28)	1.50 (1.41–1.59)	1.55 (1.46–1.63)

*Obtained by adjusting to linear regression model, using the generalized estimation equations (GEE) technique.
